# Proteasome β5i Subunit Deficiency Affects Opsonin Synthesis and Aggravates Pneumococcal Pneumonia

**DOI:** 10.1371/journal.pone.0153847

**Published:** 2016-04-21

**Authors:** Felicia Kirschner, Katrin Reppe, Nadine Andresen, Martin Witzenrath, Frédéric Ebstein, Peter-Michael Kloetzel

**Affiliations:** 1 Institut für Biochemie, Charité Universitätsmedizin Berlin, Berlin, Germany; 2 Department of Infectious Diseases and Pulmonary Medicine, Charité Universitätsmedizin Berlin, Berlin, Germany; University of Florida, UNITED STATES

## Abstract

Immunoproteasomes, harboring the active site subunits β5i/LMP7, β1i/LMP2, and β2i/MECL1 exert protective, regulatory or modulating functions during infection-induced immune responses. Immunoproteasomes are constitutively expressed in hematopoietic derived cells, constituting the first line of defense against invading pathogens. To clarify the impact of immunoproteasomes on the innate immune response against *Streptococcus pneumoniae*, we characterized the progression of disease and analyzed the systemic immune response in β5i/LMP7^-/-^ mice. Our data show that β5i/LMP7 deficiency, which affected the subunit composition of proteasomes in murine macrophages and liver, was accompanied by reduced transcription of genes encoding immune modulating molecules such as pentraxins, ficolins, and collectins. The diminished opsonin expression suggested an impaired humoral immune response against invading pneumococci resulting in an aggravated systemic dissemination of *S*. *pneumoniae* in β5i/LMP7^-/-^ mice. The impaired bacterial elimination in β5i/LMP7^-/-^ mice was accompanied by an aggravated course of pneumonia with early mortality as a consequence of critical illness during the late phase of disease. In summary our results highlight an unsuspected role for immuno-subunits in modulating the innate immune response to extracellular bacterial infections.

## Introduction

The ubiquitin proteasome system (UPS) is the primary non-lysosomal ATP-dependent protein degradation machinery in eukaryotic cells [1]. By degrading short-lived poly-ubiquitin-tagged substrates, it determines the availability of regulatory proteins and controls a large number of physiologically important cellular processes such as gene regulation and inflammatory response [2]. The 26S proteasome is the central proteolytic enzyme of the UPS. The catalytic activity is restricted to three β-subunits (β1, β2, and β5) of the 20S proteasome core particle [3]. In response to IFN-γ three alternative proteolytically active β-subunits, β1i/LMP2, β2i/MECL1, and β5i/LMP7 are incorporated into a nascent 20S proteasome generating a new sub-type, named immunoproteasome (i-proteasome) [1]. Constitutive expression of the immuno-subunits occurs in hematopoietic derived cells such as splenocytes, macrophages, and dendritic cells [2]. In comparison to standard proteasomes which are constitutively expressed in all cell types, i-proteasomes exhibit altered cleavage preferences affecting the versatility of antigen processing and the possibility to generate and present immunogenic epitopes onto MHC-class I molecules [4–6]. Therefore, β5i/LMP7-deficient mice are impaired in i-proteasome formation or efficient anti-viral and anti-bacterial immune response, as shown for CVB-3 [7] and *L*. *monocytogenes* infection [8], respectively.

However, i-proteasomes not only function by generating MHC-class I epitopes, but also possess additional immunological functions in connection with inflammatory diseases. For example, β5i/LMP7^-/-^ mice exhibit an attenuated experimental colitis with no induction of pro-inflammatory cytokines and reduced intestinal inflammation [9,10]. Moreover, recent human genetics studies, identifying missense or nonsense mutations in the PSMB8 gene encoding β5i/LMP7, support the involvement of i-proteasomes in inflammatory disorders [11]. All patients carrying mutations in the PSMB8 gene suffer from symptoms displaying recurrent fever, elevated levels of acute phase molecules, high levels of IL-6, and auto-immune abnormalities [11]. Several studies underline the role of i-proteasomes in specific pro-inflammatory signaling pathways, by controlling gene expression of immune modulators or altering the protein turnover rate of critical signaling molecules [2,9,12].

*Streptococcus pneumoniae* is estimated to cause 27% of all cases of community acquired pneumonia, being the most frequent pathogen causing lower respiratory tract infections [13]. Depending on the serotype *S*. *pneumoniae* causes pneumonia, otitis media, meningitis, and bacteremia [14]. A cohort study estimated the 28-day mortality in patients, hospitalized due to community acquired pneumonia, to 13% in South America, 9% in Europe, and 7% in North America [15]. Multiple antibiotic resistance has rapidly increased exerting 21% drug resistance to clindamycin, 39% to erythromycin, and 21% to oral penicillin according to a US study [16]. Because antibiotic resistance still spreads *S*. *pneumoniae* remains a critical pathogen [17].

The immune system employs numerous mechanisms to protect the host from an invasive pneumococcal infection. Releases of bacterial cell-wall particles, toxins, and DNA initiate activation of these defense mechanisms via pattern-recognition receptors (PRRs) [18]. They recognize bacterial components containing conserved motifs that are referred to as pathogen-associated molecular patterns (PAMPs) [19]. PRRs such as TLRs stimulate the production of NF-κB and AP-1 dependent immune modulating components via the MyD88, TRIM/TRAF, and MAP kinase pathway resulting in activation of innate immune defenses [18]. Alveolar macrophages represent the first phagocytic defense and are essential in initiating and coordinating the innate immune response to infection [20]. In addition to phagocytosis and endosomal clearance of *S*. *pneumoniae*, they rapidly produce large amounts of pro-inflammatory cytokines as well as reactive oxygen species. Alveolar macrophages are activated by PAMPs but also by cytokines, complement, and antibody complexes [21], and finally contribute to the resolution of inflammation in lung tissue by clearing apoptotic cells [22]. In established pneumonia, neutrophils become the major cells eliminating bacteria. Chemokines such as CXCL-1 stimulate their chemotaxis [23]. If alveolar defense mechanisms are overwhelmed, invasion of *S*. *pneumoniae* into the bloodstream takes place. Clearance of pneumococci from circulation strongly depends on opsonization enhancing bacterial phagocytosis by leukocytes [24]. Pentraxins are cytokine-inducible soluble PRRs and part of the humoral innate immune system [25]. They opsonize bacteria and promote phagocytosis by macrophages and neutrophils [26]. They include the short pentraxin C reactive protein (CRP), which is systemically produced in liver during the acute phase response and the long pentraxin PTX-3 [27]. PTX-3 is produced in various cell types, with macrophages being one of the major sources. Pentraxins interact with opsonizing components (C1q, MBL, and Ficolin) of the classical and lectin activation pathway of the complement system, thereby amplifying its effector functions [28].

Using β5i/LMP7^-/-^ mice, we studied the impact of i-proteasomes on the innate immune response against *S*. *pneumoniae*. Our experiments revealed that β5i/LMP7 deficiency alters the composition of proteasomes in murine macrophages and liver, which is accompanied by reduced gene transcription of immune modulating molecules. Reduced opsonin expression and impaired bacterial elimination in β5i/LMP7^-/-^ mice is accompanied by a more severe case of pneumonia with early mortality in consequence of critical illness during the late phase of disease. Taken together, our findings highlight an unexpected role for i-proteasomes in modulating the innate immune response to extracellular bacterial infections.

## Material and Methods

### Cell culture experiments

Primary cells were obtained from WT and β5i/LMP7^-/-^ C57BL/6 (J) mice. The protocol was approved by the Committee on the Ethics of Animal Experiments of Berlin State authorities (Landesamt für Gesundheit und Soziales Berlin, approval ID: T0371/11). Cell lines (L929, Raw 264.7) were kindly provided by Prof. Bastian Opitz (Charité Universitätsmedizin Berlin, Berlin, Germany). Cells were stimulated with LPS (Sigma Aldrich, Hamburg, Germany) and epoxomicin (Merck, Darmstadt, Germany).

### Bacterial strains

*Streptococcus pneumoniae* strain *D39Δcps (*serotype 2 carrying a deletion mutation in the polysaccharide capsule gene) was used in cell culture experiments and *S*. *pneumoniae* strain PN36 (serotype 3, NCTC7978) was used to induce pneumonia in *in vivo* experiments. Bacteria were grown in THY medium (30% Todd-Hewitt-Bouillion (BD Bioscience, San Jose, CA, USA), 0.5% Yeast (BD Bioscience, San Jose, CA, USA) at 37°C and 5% CO_2_ until they reached a phase of logarithmic growth. For heat-inactivation, bacteria where incubated at 56°C for 1 h while shaking.

### Generation and cultivation of bone marrow derived macrophages (BMM)

Bone marrow was isolated by flushing the opened cavity of femur and tibia with RPMI (Biochrom, Berlin, Germany). A single cell suspension was prepared, and incubated over night by 37°C and 5% CO_2_ in RPMI and 10% FCS (Biochrom, Berlin, Germany) in a cell culture dish. Non-adherent cells were collected and differentiated in RPMI with 30% L929 supernatant, containing m-CSF, for 9 days.

### Phagocytosis assay

BMM were covered with ice-cold PBS containing heat-inactivated D39Dcps-PKH67 (MOI 25) and placed on ice for 15 min. Except for the negative control cells, which remained on ice, macrophages were incubated at 37°C for up to 120 min. Finally, cells were placed on ice and D39Dcps-PKH67 were removed. Cells were washed with ice-cold PBS and harvested. The florescent intensity of internalized D39Dcps-PKH67 was measured by flow cytometry. Heat-inactivated D39Dcps were fluorescently labeled with PKH67 using PKH67 linker kit (Sigma Aldrich, Hamburg, Germany).

### Gentamicin protection assay

6×10^5^ or 1×10^6^ BMM were seeded on a 12-or 6-well plate culture dish and co-cultivated with D39Dcps (MOI 10) without antibiotics at 37°C for 30 min or 4 h. In order to remove remaining extracellular bacteria, cells were washed with pre-warmed PBS and cultivated further with gentamicin (300 mg/ml or 20 mg/ml) (Lonza, Aboise, France). After an incubation period of several hours, cells were washed with PBS and lysed by adding PBS with 0.02% Triton X (Sigma Aldrich, Hamburg, Germany). Serial dilutions of the supernatant were plated on blood agar (BD Bioscience, San Jose, CA, USA) and the CFUs were determined.

### Indirect measurement of NO

Macrophages were stimulated and the nitrite content of the supernatant determined, using the Griess Reagent kit for nitrite determination (Molecular Probes, Darmstadt, Germany).

### Animal infection experiments

Mice were kept at the animal facilities of the Charité-Universitätsmedizin Berlin, according to the European and Berlin State guidelines for animal welfare. Mice were bred in a heterozygous mating. All animal experimental protocols were approved by the Committee on the Ethics of Animal Experiments of Berlin State authorities (Landesamt für Gesundheit und Soziales Berlin, approval ID: G0073/11). All analyzed groups contained female littermates with an age of 8–11 weeks exclusively. For infection, mice were anesthetized by intraperitoneal (i.p.) application of ketamine (80 mg/kg) and xylazine (25 mg/kg) (both Cp pharma, Burgdorf, Germany) and transnasally inoculated with 7.5×10^4^ or 5×10^6^ CFU *S*. *pneumoniae* (PN36) in 20 μl sterile phosphate-buffered saline (PBS) as described previously [29]. In uninfected mice, 20 μl PBS/mouse was transnasally applied as control. Disease severity of infected mice was monitored and documented every 12 h. Survival was recorded for 10 days post infection. Mice were humanely sacrificed by exsanguination via the *Vena cava caudalis* after i.p. injection of ketamine (160 mg/kg body weight) and xylazine (75 mg/kg), when they reached at least one of the predefined humane endpoints ([(i) body temperature <30°C, (ii) body weight loss ≥ 20%, (iii) cumbersome breathing, (iv) accelerated breathing in combination with staggering, pain or paleness]). No unexpected cell death was observed in these experiments. For the analysis of blood leukocytes and bacterial burden in lungs and blood 24 or 48 h post infection, anesthetized (160 mg/kg mg ketamine, 75 mg/kg xylazine) mice were tracheotomised and heparinized. Blood was taken via the *Vena cava caudalis* and kept on ice in EDTA-coated tubes. For determination of bacterial burden in lungs, these were dissected without trachea and main bronchi and minced by pressing the tissue through a 100 μm cell strainer. Serial dilutions of samples were plated on blood agar, and CFUs were determined.

### Cytokine multiplex assay

Chemokine concentrations in plasma of infected mice were measured by using the Bio-Plex Pro Mouse Cytokine Group I Assay (Bioplex^®^, BioRad, Hercules, CA) and the multiplex working station, provided by the Deutsches Rheuma-Forschungszentrum (DRFZ) Berlin, Germany.

### Flow cytometry

For analysis of the cellular immune status, total blood leukocytes were counted by fluorescence-activated cell sorter (FACS) analysis (FACS Calibur; BD Biosciences, Heidelberg, Germany) using BD TruCount Tubes (BD Bioscience, San Jose, CA, USA), forward vs. side scatter characteristics and antibody staining with CD45-PerCP (clone 30-F11; BD Bioscience, San Jose, CA, USA), GR-1-PE (clone RB6–8C5; BD Bioscience, San Jose, CA, USA).

### Bronchoalveolar lavage in vivo

Mice transnasally infected with 5×10^6^ CFU PN36 were anesthetized 48 h post infection with ketamine (160 mg/kg body weight (BW)) and xylazine (75 mg/kg BW), tracheotomized and ventilated as previously described [30]. After heparinization, blood was drawn from the *Vena cava caudalis*. Lungs were flushed with sterile 0.9% saline *via* the pulmonary artery. Afterward, bronchoalveolar lavage (BAL) was performed twice using 800 μl PBS containing protease inhibitor each time (Roche, Mannheim, Germany) After centrifugation of both BAL fluids at 800 × g, the supernatants were separately snap frozen. Supernatant of the first BAL was used for protein and albumin analysis. The amount of murine serum albumin in plasma and BAL fluid was measured by ELISA according to manufacturer’s protocol (Bethyl, Montgomery, TX, USA). Protein quantification was performed using BCA protein assay kit (Thermo Fisher Scientific, Waltham, MA USA).

### Serological Analysis

The enzyme activity of ALAT and LDH in serum of ill animals was measured by the Institute of Veterinary Medicine and Diagnostics, Berlin, Germany.

### Immuno-blotting

Equal amounts of protein extracts were separated by SDS-PAGE and transferred onto nitrocellulose membranes (Merk, Darmstadt, Germany). I-proteasome subunits were analyzed by immuno-blotting using anti-mouse anti-β5i/LMP7, anti-β5, anti-β1i/LMP2 (Abcam, Cambridge, UK) and anti-β1, anti-β2i/MECL-1, anti-α4 (self-made), and AP1 components, anti-(p)-cJun, anti-(p)-cFos, anti-cFos, anti-(p)-ATF-2, anti-ATF-2 (Cell signaling, Leiden, Netherlands), and anti-cJun (Santa Cruz Biotechnologie, Dallas, Texas, USA) antibodies combined with a secondary polyclonal mouse-anti-rabbit-IgG antibody conjugated to horseradish peroxidase (Dianova, Hamburg, Germany). Anti-β-actin antibody (Cell signaling, Leiden, Netherlands) was used as loading control.

### 20S purification and 2D Gel analysis

One billion BMM were pestled in TEAD buffer, using a douncer, and centrifuged for 60 min at 13.000 rpm and 4°C. The resulting supernatant was used for the purification of 20S proteasomes, as previously described [31]. The purified 20S proteasome was precipitated in ethanol (96%) overnight at -20°C. Precipitated 20S proteasomes were centrifuged for 60 min at 13.000 rpm and 4°C, and pellets was washed with ethanol (70%). The proteasomes were centrifuged again for 30 min at 13.000 rpm and 4°C, pellets were dried, and the 2D electrophoresis was carried out by Protalys GmbH, Berlin, Germany.

### Quantitative real-time RT-PCR

Total RNA was extracted using Trizol-Chlorophorm extraction (Invitrogen, Darmstadt, Germany) from cells and pestled tissue followed by reverse transcription using cDNA generation kit (Roche, Penzberg, Germany). The cDNA was used for PCR amplification with the light cycler instrument (Rotor gene 3000 cycler, Corbett Research) and a TaqMan Real Time Mastermix (Applied Biosystems, Darmstadt, Germany) with the following TaqMan probes (Applied Biosystems, Darmstadt, Germany): Saa1 (Mm01545399_m11), Mbl2 (Mm0087623_m1), C1qa (Mm00432142), Ptx3 (Mm00477268_m1), Fcna (Mm00484287_m1), PAFR (Mm02621061_m1) and IL-1-β (Mm00434228_m1). Gene expression was normalized to the housekeeping gene HPRT1 (Mm01545399_m1) by means of the ΔCt method.

### Statistics

The statistical significance was determined by using the Mann-Whitney U Test for *in vivo* as well as *ex vivo* data and by using the Student t-test for *in vitro* generated data. All statistical analyses were performed using GraphPad Prism Software (version 4.03) (GraphPad, San Diego, CA, USA). Statistical significance was achieved when p<0.05; *p<0.05, **p<0.01, ***p<0.001.

## Results

### β5i/LMP7 deficiency aggravates clinical signs of pneumococcal pneumonia

**Fig 1:**
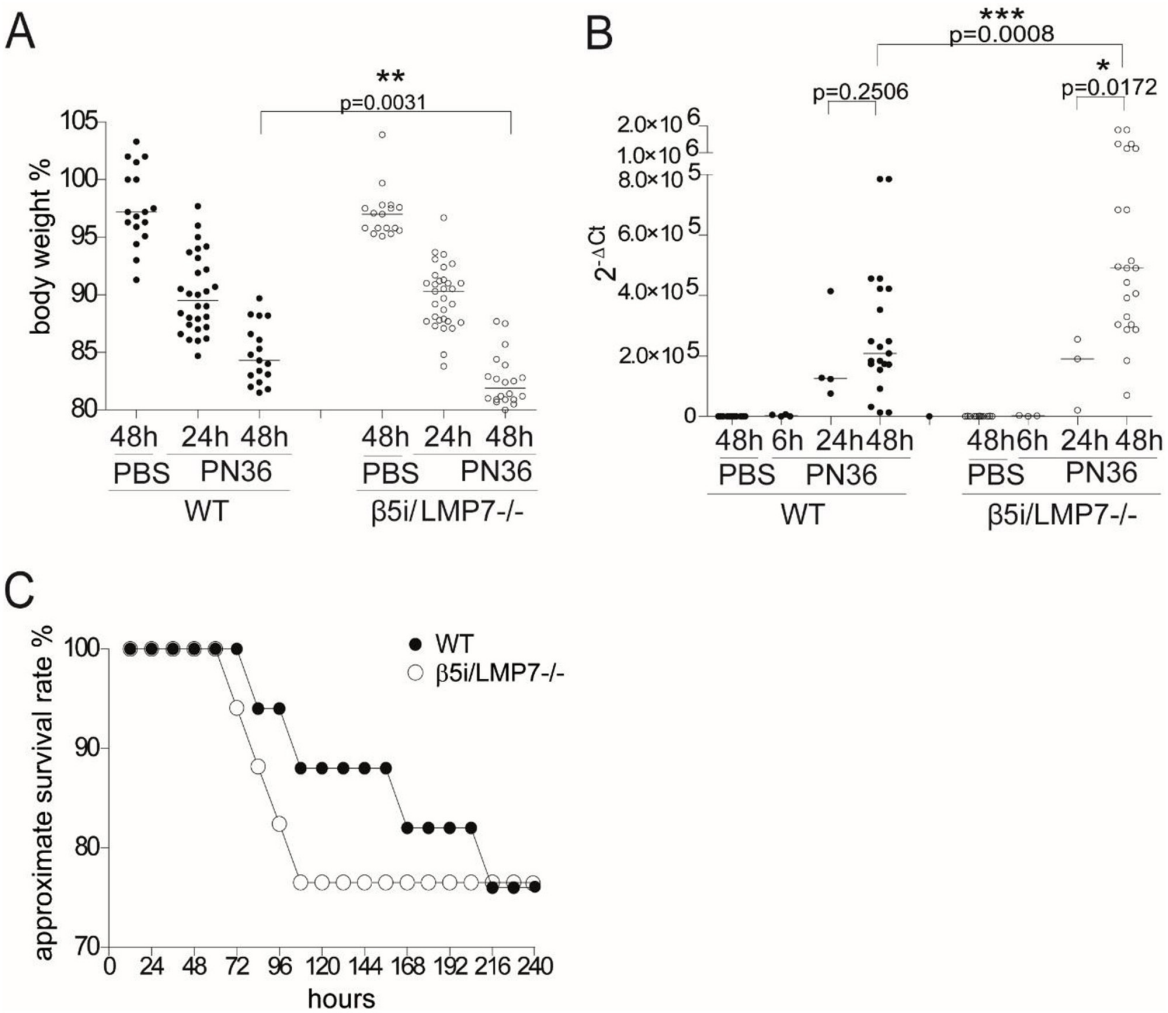
β5i/LMP7^-/-^ mice suffer more than WT mice from severe pneumonia. (A) Monitoring of body weight 24 h and 48 h after transnasal application of 5×10^6^ CFU PN36/mouse (each group with n = 12–29). (B) Analysis of mRNA expression of the acute phase protein SAA in liver after transnasal application of 5×10^6^ CFU PN36/mouse, performed by RT and Real Time PCR (each group with n = 3–14) (statistical analysis by Mann Whitney U Test. *p<0.05; **p<0.01; ***p<0.001). (C) Assessment of the approximate survival rate for up to 10 days upon transnasal application of 7.5x10^4^ CFU PN36/mouse (each group with n = 17).

To illustrate the role of i-proteasomes in host defense during *S*. *pneumoniae* infection, we examined the impact of β5i/LMP7 deficiency on the clinical course of pneumococcal pneumonia. Infected animals developed a hypodynamic state, associated with a gradual loss in body weight from 24 h to 48 h of infection (Fig 1A). The body weight loss was significantly stronger in β5i/LMP7^-/-^ compared to WT animals. High levels of acute phase proteins accurately correlate with the presence and severity of infection, and thus, are commonly used as diagnostic marker for the extent and outcome of infection [32]. The major acute phase protein, synthesized in mice, is serum amyloid A (SAA) [33]. The expression of *saa1* started at 24 h of infection and increased further over time during the late phase of pneumonia, in both mouse strains, though much stronger and only significant in β5i/LMP7^-/-^ mice (Fig 1B). In order to investigate whether β5i/LMP7 is crucial for survival of pneumococcal pneumonia, β5i/LMP7^-/-^ and WT mice were monitored for their ability to resist to infection (Fig 1C). Although no difference in the overall approximate survival rate was observed between WT and β5i/LMP7^-/-^ mice, mice deficient in the β5i/LMP7 subunit and hence in fully functional i-proteasomes died at earlier time points with an enhanced mortality during the first 108 h of infection, showing an approximate death rate of about 12% in WT versus about 23.5% in β5i/LMP7^-/-^ mice. Altogether, the aggravated loss in body weight, the more pronounced expression of *saa1* and the premature mortality pointed to an aggravated course of pneumonia in β5i/LMP7^-/-^ mice especially during the later phase of the disease (48 h post infection).

### β5i/LMP7^-/-^ but not WT mice develop sepsis

**Fig 2:**
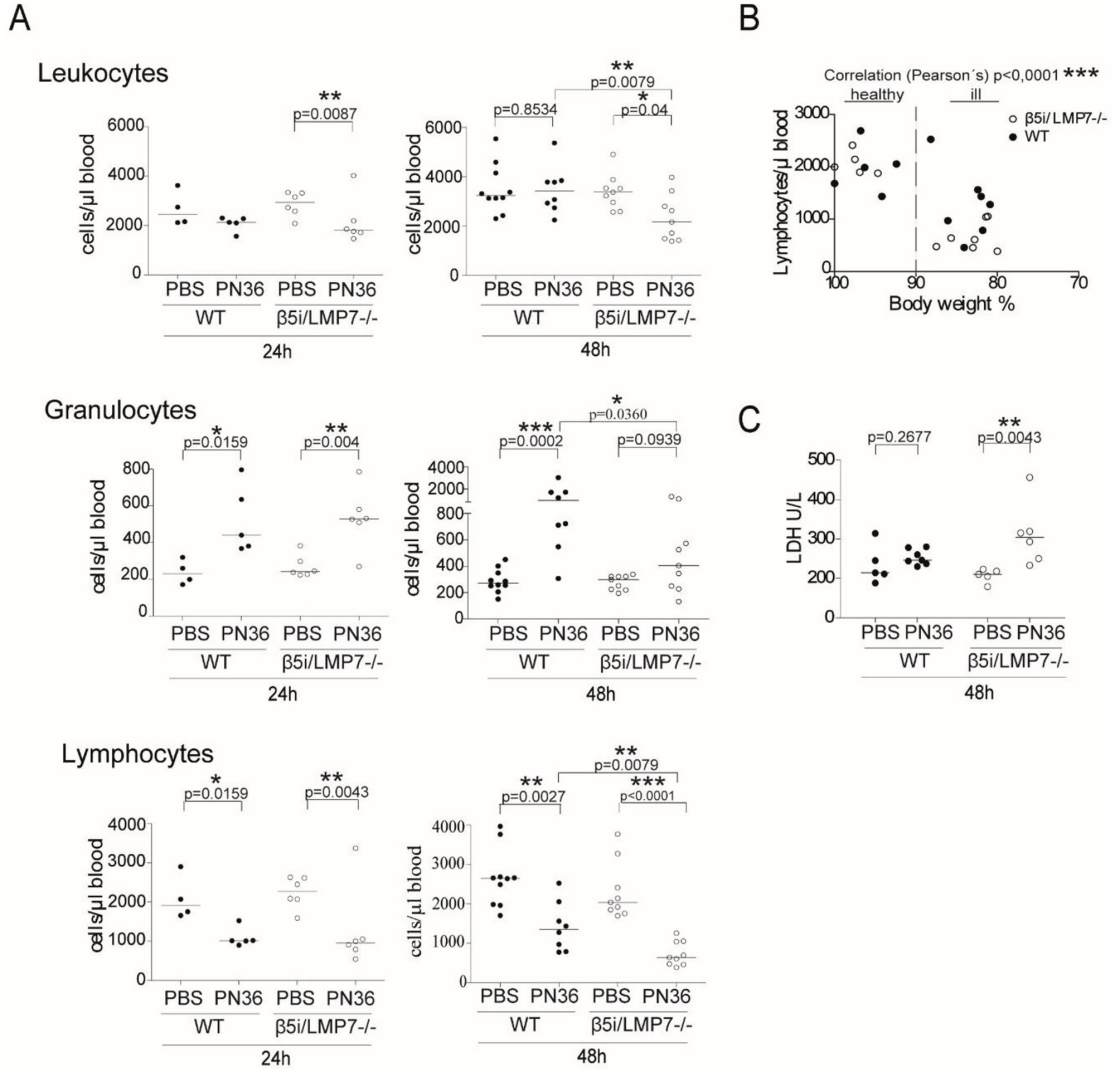
β5i/LMP7^-/-^ mice suffer from a systemic inflammatory response. **(A)** Determination of blood leukocytes, analyzed by flow cytometry 24 h and 48 h after application of 5×10^6^ CFU PN36/mouse. As control, 20 μl PBS/mouse was applied transnasally (each group with n = 4–10; statistical analysis by Mann Whitney U Test. *p<0.05; **p<0.01; ***p<0.001). (B) Correlation of lymphopenia and disease severity, indicated by body weight. Lymphocytes were detected by FACS analysis 48 h after application of 5×10^6^ CFU PN36/mouse (each group with n = 9–10, statistical analysis by Pearson’s Correlation. ***p<0.001). (C) Documentation of tissue damage 48 h post infection by measuring LDH activity in serum of mice, transnasally infected with 5×10^6^ CFU PN36/mouse (each group with n = 6–7) (statistical analysis by Mann Whitney U Test. *p<0.05; **p<0.01; ***p<0.001).

To validate the influence of β5i/LMP7 deficiency on the severity of the systemic inflammatory response, we next quantified and discriminated the levels of circulating immune cells 24 h and 48 h post infection (Fig 2A). A significant recruitment of granulocytes was detected in both mouse strains 24 h post infection that further augmented to an approximate 3.5-fold increase in WT animals. In contrast, in β5i/LMP7^-/-^ mice, the recruitment of granulocytes diminished to an approximate 1.3-fold difference at 48 h of infection. Throughout the whole experiment, levels of leukocytes remained unaffected in WT animals after infection, whereas their numbers significantly declined in β5i/LMP7^-/-^ mice. At 24 h of infection, the amount of circulating leukocytes decreased by about 35% compared to uninfected control littermates. After 48 h of infection β5i/LMP7^-/-^ mice exhibited significantly less leukocytes compared to WT animals. The prominent leukopenia was established by a significant lymphopenia. At 24 h of infection, reduced levels of circulating lymphocytes were detected in both mouse strains. However, at the 48 h time point, the total number of lymphocytes decreased even further in β5i/LMP7^-/-^ animals, establishing a significant difference between infected WT and β5i/LMP7^-/-^ animals. A correlation analysis between body weight, as indicator of health, and amount of lymphocytes showed its association with severity of disease (Fig 2B). A severe sepsis is defined by a severe systemic inflammatory response that is accompanied by peripheral tissue injuries [34]. In order to assess tissue damage, we evaluated serum levels of the enzyme lactate dehydrogenase (LDH), which is generally released as a result of cell destruction. Only β5i/LMP7^-/-^ animals exhibited substantial LDH serum levels after 48 h of infection (Fig 2C). Besides, the strong recruitment of granulocytes at 24 h of infection suggested a prominent systemic immune reaction. As indicated by a depletion of circulating leukocytes (lymphocytes, granulocytes), this was accompanied by relative systemic immune suppression at 48 h of infection in β5i/LMP7^-/-^ animals. Thus β5i/LMP7 deficiency and hence i-proteasome deficiency resulted in a progressed systemic inflammatory response.

**Fig 3:**
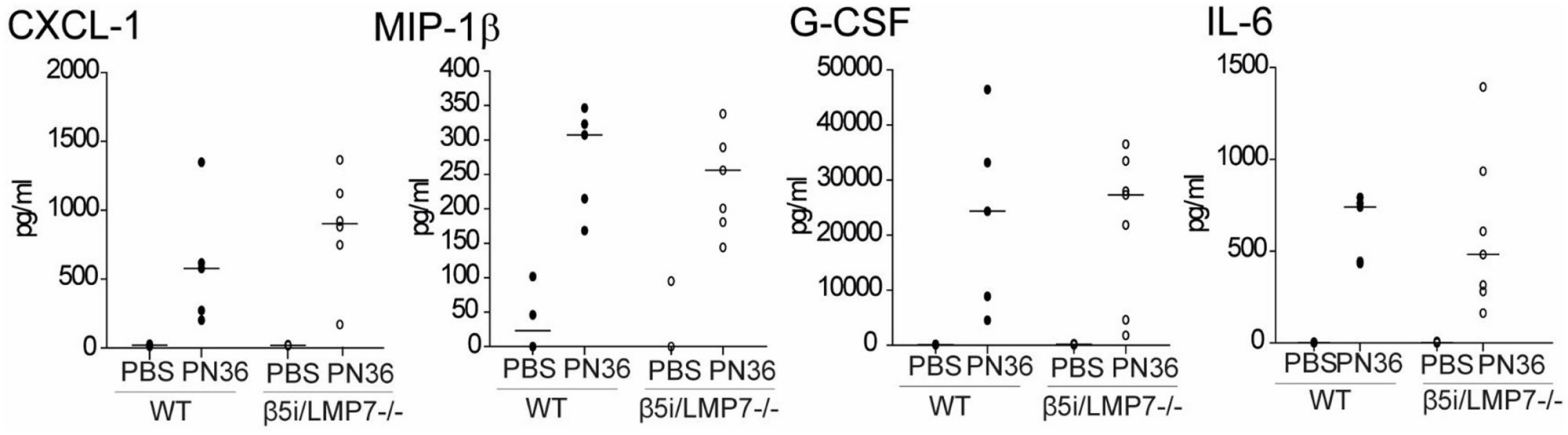
Systemic levels of chemokines increase after infection, but do not differ between WT and β5i/LMP7^-/-^ mice. Determination of chemokines in plasma, obtained 48 h after transnasal application of 5×10^6^ CFU PN36/mouse, and measured by cytokine multiplex assay (each group with n = 5–7).

Because CXCL-1 and G-CSF plasma levels, which promote development and recruitment of granulocytes, were almost identical in both mouse strains, we reasoned that the lack of chemokines was not the cause for the reduced neutrophil enrolment (Fig 3).

### β5i/LMP7 deficiency aggravates bacteremia during the late phase of pneumonia

**Fig 4:**
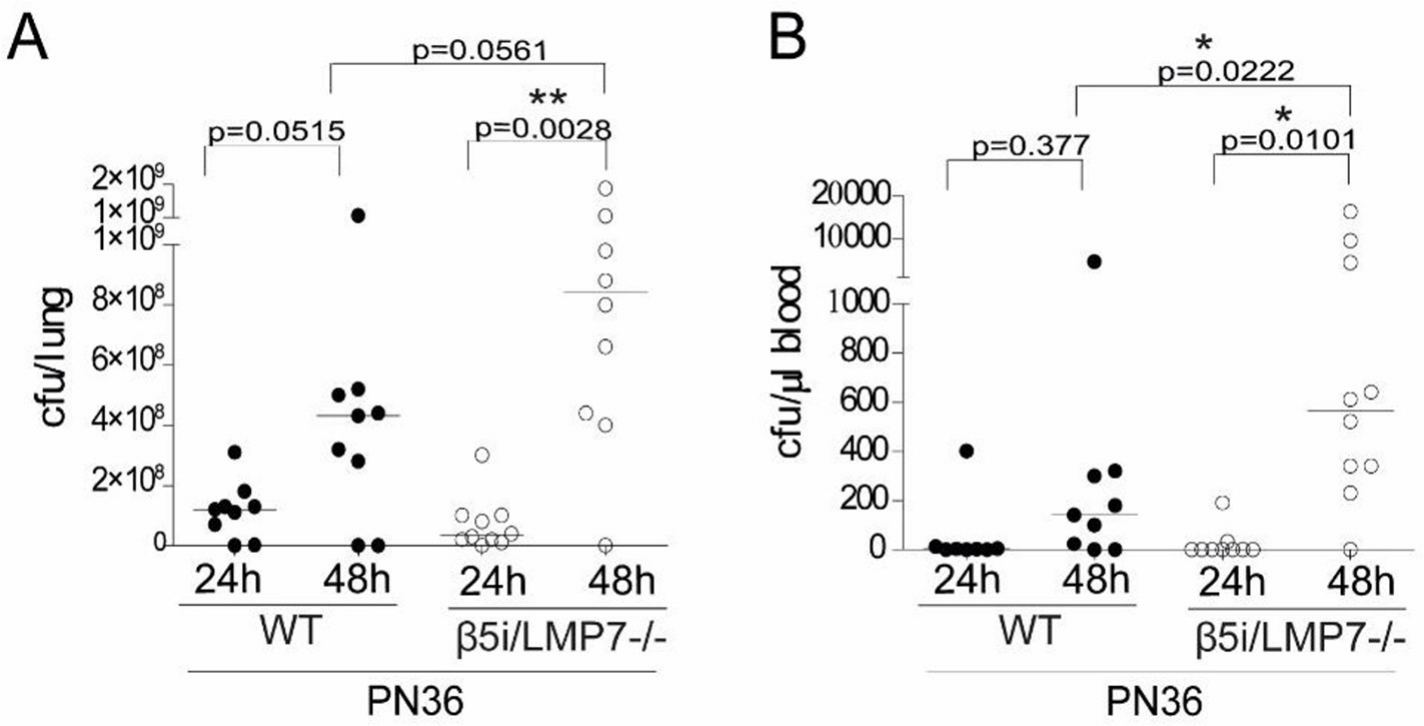
β5i/LMP7 deficiency is accompanied by an elevated bacterial load. Determination of bacterial load 24 h and 48 h post infection in (A) lung and (B) blood of mice, transnasally infected with 5×10^6^ CFU PN36/mouse (each group with n = 9–10; statistical analysis by Mann Whitney U Test. *p<0.05).

We next assessed the extent of bacterial growth in lung and determined the magnitude of bacterial dissemination. Within the first 24 h of infection a considerable proliferation of *S*. *pneumoniae* was documented in lungs of both mouse strains (Fig 4A) reaching extremely high numbers. Between 24 h and 48 h of infection, proliferation continued with β5i/LMP7^-/-^ mice exhibiting a higher bacterial load than infected WT animals. However, due to high variations this difference did not reach statistical significance. Indeed, hemocultures started to become positive after 24 h of infection and after 48 h of infection nearly 100% of all WT and β5i/LMP7^-/-^ animals established bacteremia (Fig 4B). On the contrary, during the late phase of pneumonia, at 48 h of infection, β5i/LMP7^-/-^ mice showed an exacerbated bacterial dissemination, as evidenced by a significantly higher bacterial load in blood compared to WT animals.

Because the airway epithelial platelet-activating factor receptor (PAFR) has been reported to facilitate invasion of *S*. *pneumoniae* to the bloodstream by interacting with bacterial phosphorylcholine [35,36], we next asked whether its expression was altered in β5i/LMP7^-/-^ animals. As shown in S1 Fig, the up-regulation of PAFR upon infection was significantly less pronounced in β5i/LMP7^-/-^ mice than in their WT counterparts. These data therefore suggest that the higher number of pneumococci observed in the blood system of β5i/LMP7-deficient mice is unlikely to be due to increased invasion of *S*. *pneumoniae* through PAFR.

**Fig 5:**
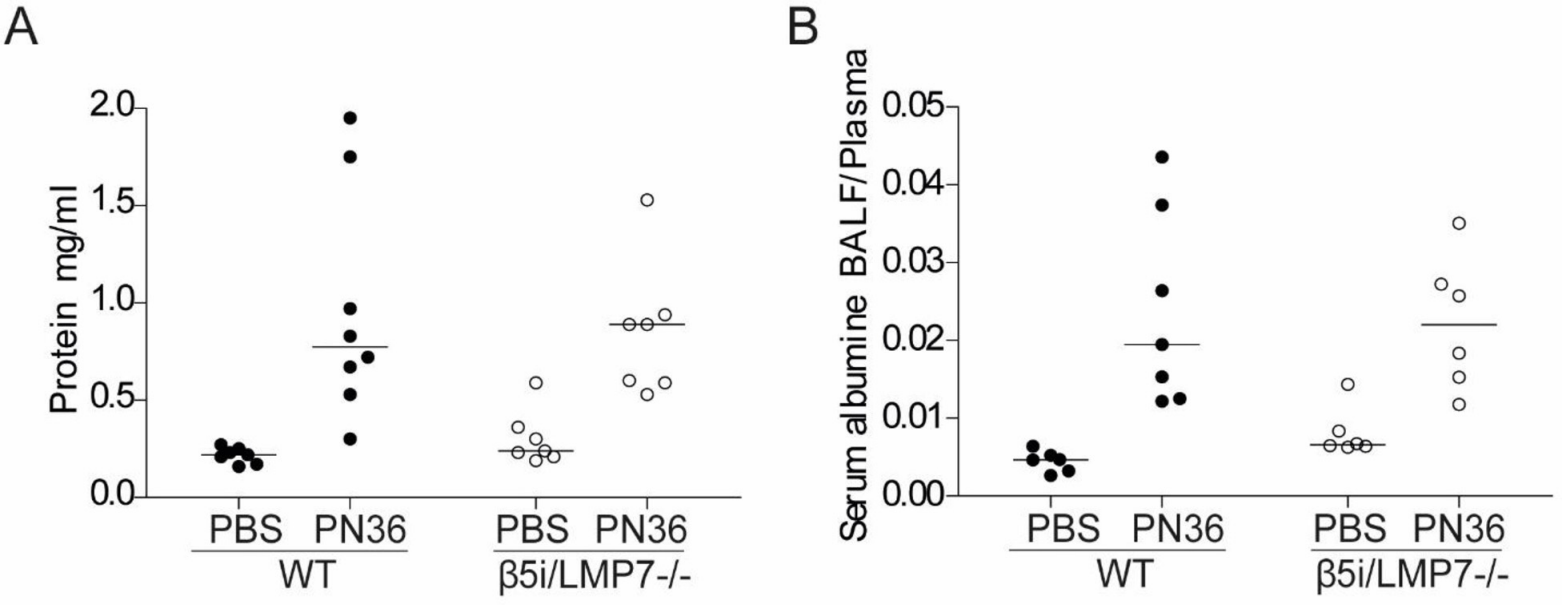
Pulmonary infection provokes oedema in lungs of β5i/LMP7^-/-^ and WT mice. **(A)** Detection of endo-epithelial cell damage, assessed by determining protein concentration in BAL fluid, obtained 48 h after transnasal application of 5×10^6^ CFU PN36/mouse, and performed by BCA (each group with n = 7–8). **(B)** Evaluation of endo-epithelial barrier disruption by measuring concentrations of serum albumin in BAL fluid, obtained 48 h after transnasal application of 5×10^6^ CFU PN36/mouse by ELISA (each group with n = 7–8).

Pneumococcal dissemination is known to be facilitated by serious damage of the alveolar endo-epithelial barrier. However, detection of endo-epithelial cell damage as assessed by determining protein concentration in BAL fluid 48 h after transnasal application of 5×10^6^ CFU PN36/mouse and evaluation of endo-epithelial barrier disruption by measuring concentrations of serum albumin in BAL fluid 48 h after transnasal application of 5×10^6^ CFU PN36/mouse did not provide any evidence for an aggravated endo-epithelial disruption due to β5i/LMP7 deficiency, as indicated by the protein and serum albumin content in BAL fluid of infected animals (Fig 5A and 5B).

### The efficiency to kill *S. pneumoniae* is unaffected in β5i/LMP7^-/-^ leukocytes

**Fig 6:**
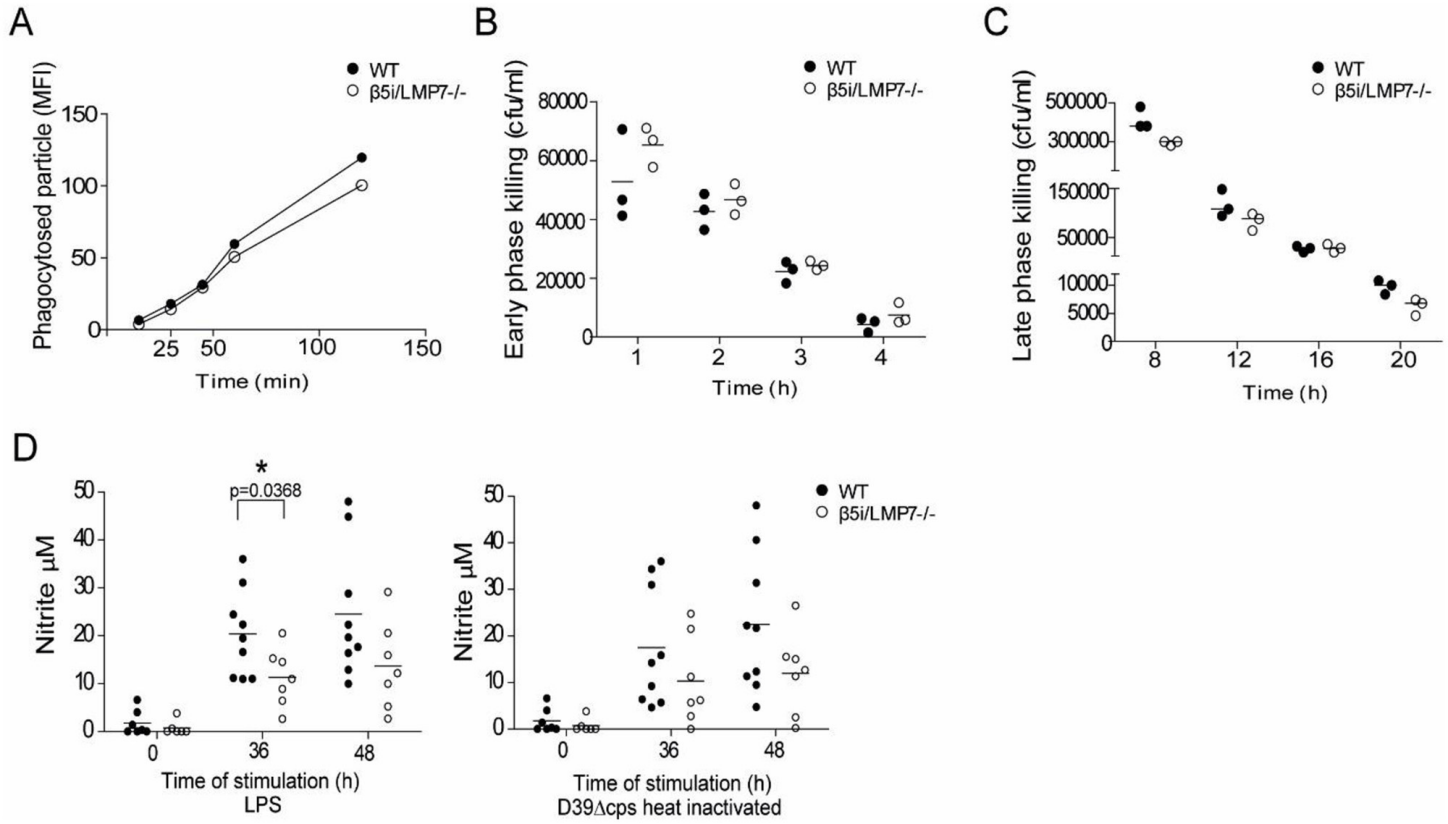
Efficiency of bacterial killing is unaltered in β5i/LMP7^-/-^ leukocytes. (A) Analysis of phagocytosis of fluorochrome-labelled particles by BMM, determined by flow cytometry (each group with n = 3). (B) Analysis of the intracellular early phase of killing and (C) late phase of killing of D39Δcps by BMM. Therefore, BMM were co-cultivated with D39Δcps MOI 10, cells were lysed, and cfu/ml were determined (each group with n = 3). (D) Analysis of NO secretion by WT and β5i/LMP7^-/-^ BMM, stimulated either with LPS (1 μg/ml) or heat-inactivated D39Δcps lysate (MOI 50). NO concentrations were photometrically determined in the supernatant by Griess-Reagent (each group with n = 7; statistical analysis by student´s t-test. * p<0.05).

Because β5i/LMP7^-/-^ mice exhibited a higher bacterial load in blood, we investigated the impact of β5i/LMP7 deficiency on leukocyte function and their ability to eliminate *S*. *pneumoniae*. To analyze phagocytosis efficiency, bone marrow derived macrophages (BMM) were incubated with fluorochrome-labelled heat-inactivated pneumococci. FACS analysis revealed no difference in the amount of WT and β5i/LMP7^-/-^ BMM, having incorporated these particles (Fig 6A). Co-cultivating macrophages with pneumococci showed that β5i/LMP7 deficiency had no impact on intracellular killing efficiency. Macrophages of both WT and β5i/LMP7^-/-^ mice effectively eliminated *S*. *pneumoniae* in the early and late phase of intracellular killing (Fig 6B and 6C). Extracellular killing is facilitated by reactive nitrogen species, mainly nitric oxide (NO), which is for example produced by macrophages expressing iNOS. Mice deficient for iNOS are highly susceptible to systemic infection and suffer from increased bacteremia [37]. To investigate the capacity of WT and β5i/LMP7^-/-^ BMM of producing NO, we stimulated BMM with LPS or heat-inactivated *S*. *pneumoniae*, and determined the production of reactive nitrogen species indirectly by measuring NO_2_^-^ levels in supernatant. At 36 h and 48 h of stimulation, elevated NO_2_^-^ levels were detected in both mouse groups. However, β5i/LMP7^-/-^ BMM accumulated less NO_2_^-^ compared to WT BMM upon stimulation (Fig 6D), suggesting that β5i/LMP7 deficiency impairs NO production. However, it was not possible to establish a relationship between decreased NO release and bacterial counts in tissue or blood, since no NO_2_^-^ level above the background value was detected in BAL fluid of WT or β5i/LMP7^-/-^ mice (data not shown).

### β5i/LMP7 deficiency diminishes expression of opsonizing molecules

**Fig 7:**
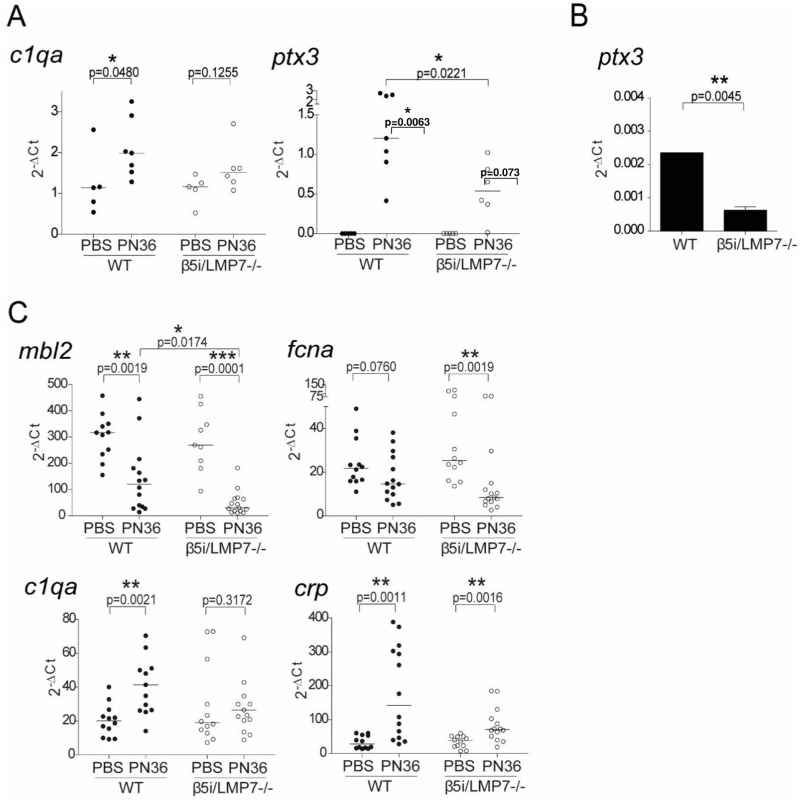
β5i/LMP7^-/-^ mice exhibit a diminished expression of opsonizing molecules in lung, liver and macrophages. (A) Gene expression analysis of lungs, 48 h after transnasal application of 5×10^6^ CFU PN36/mouse, performed by RT and Real Time PCR (each group with n = 5–6; statistical analysis by Mann Whitney U Test. *p<0.05). (B) Gene expression analysis of BMM co-cultivated with D39Δcps (MOI 10) for 4 h, performed by RT and Real Time PCR (each group with n = 4, illustrated experiment is representative for 4 individual replicates; showing mean± SD; statistical analysis by student’s t-test **p<0.01). (C) Gene expression analysis of liver 48 h after transnasal application of 5×10^6^ CFU PN36/mouse, performed by RT and Real Time PCR (each group with n = 12–14; statistical analysis by Mann Whitney U Test. *p<0.05; **p<0.01).

Because bacterial opsonization results in facilitated pathogen recognition, enhanced phagocytosis, and elimination, we next sought to assess the expression of various opsonizing molecules in lung and liver of infected animals. In lungs, we examined mRNA expression of collectins and pentraxins, including C1q (*c1qa*), and PTX-3 (*ptx3*) (Fig 7A). At 48 h of infection, the expression of *c1qa* and *ptx3* was significantly induced in WT animals. By contrast, β5i/LMP7^-/-^ mice hardly exhibited any up-regulation of these genes. These *in vivo* data were confirmed by *in vitro* experiments, in which WT and β5i/LMP7^-/-^ macrophages were co-cultivated with *S*. *pneumoniae*. *Ptx3* expression was significantly induced upon infection in both mouse groups, although to a substantially smaller extent in β5i/LMP7^-/-^ macrophages (Fig 7B). Because β5i/LMP7^-/-^ mice suffered from enhanced bacterial growth in blood, we also investigated mRNA expression of circulating collectins, ficolins, and pentraxins, including mannose binding lectin MBL (*mbl2*), c-reactive protein CRP (*crp*), C1q (*c1qa*), and Ficolin A (*fcna*), in the liver of infected WT and β5i/LMP7^-/-^ mice (Fig 7C).

**Fig 8:**
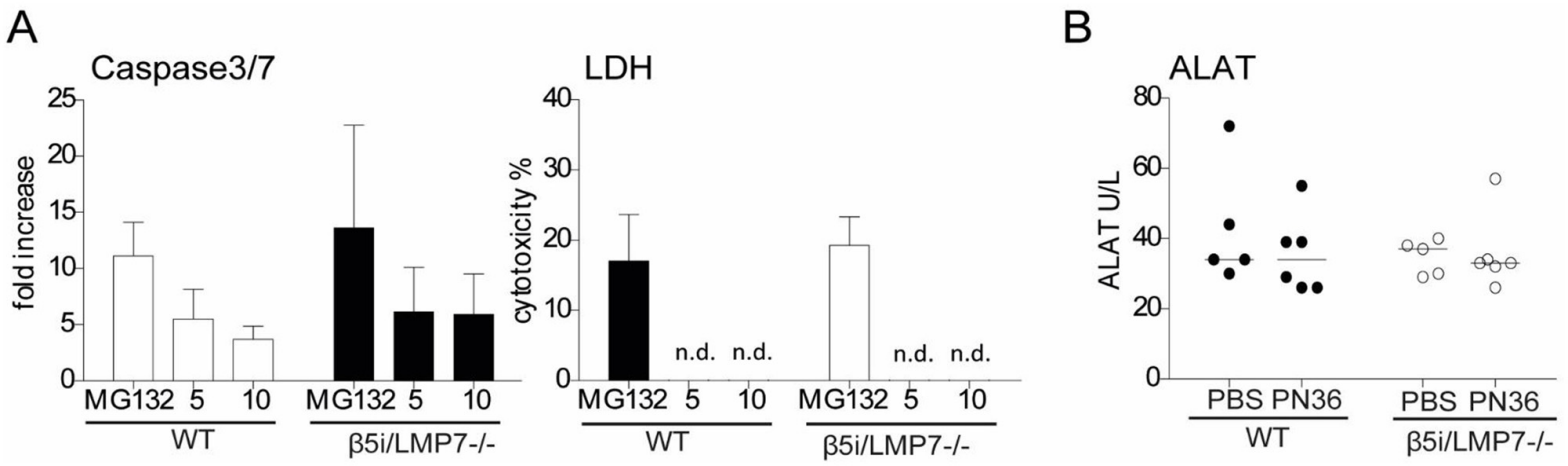
β5i/LMP7 deficiency has no apoptotic or cytotoxic effect upon infection. **(A)** Analysis of the activity of caspase 3/7 in cell lysate and LDH in supernatant of stimulated BMM. They were co-cultivated with D39Δcps (MOI 10) for 4 h, or treated with 10 μM MG132 for 24 h as positive control (each group with n = 4). **(B)** Analysis of potential liver damage, 48 h post infection, by measuring ALAT activity in serum of mice transnasally infected with 5×10^6^ CFU *PN36*/mouse (each group with n = 5–6).

At 48 h of infection a significant decline in MBL mRNA levels was detected, which was significantly more pronounced in β5i/LMP7^-/-^ mice. Similarly, *fcna* expression was also significantly down-regulated only in β5i/LMP7^-/-^ animals. In contrast, expression of C1q mRNA hardly increased in β5i/LMP7^-/-^ mice but was up-regulated in WT animals. CRP mRNA levels were significantly increased in both mouse strains, albeit to a weaker extent in β5i/LMP7^-/-^ animals. The reduced CRP expression was not the consequence of low cytokine stimulation because IL-6 levels were at equal levels in WT and β5i/LMP7^-/-^ mice (Fig 3). Furthermore, analysis of the activity of caspase 3/7 in cell lysate and LDH in supernatant of stimulated BMM that were co-cultivated with D39Δcps for 4h or treated with 10 μM MG132 for 24 h as positive control (Fig 8A) as well as analysis of potential liver damage, 48 h post infection, by measuring ALAT activity in serum of mice transnasally infected with 5×10^6^ CFU PN36/mouse (Fig 8B) revealed that deficiency in β5i/LMP7 did not exacerbate inflammation-induced apoptosis or cytotoxicity in liver or macrophages.

### β5i/LMP7 deficiency alters proteasome subunit composition independent of infection in liver and macrophages

**Fig 9:**
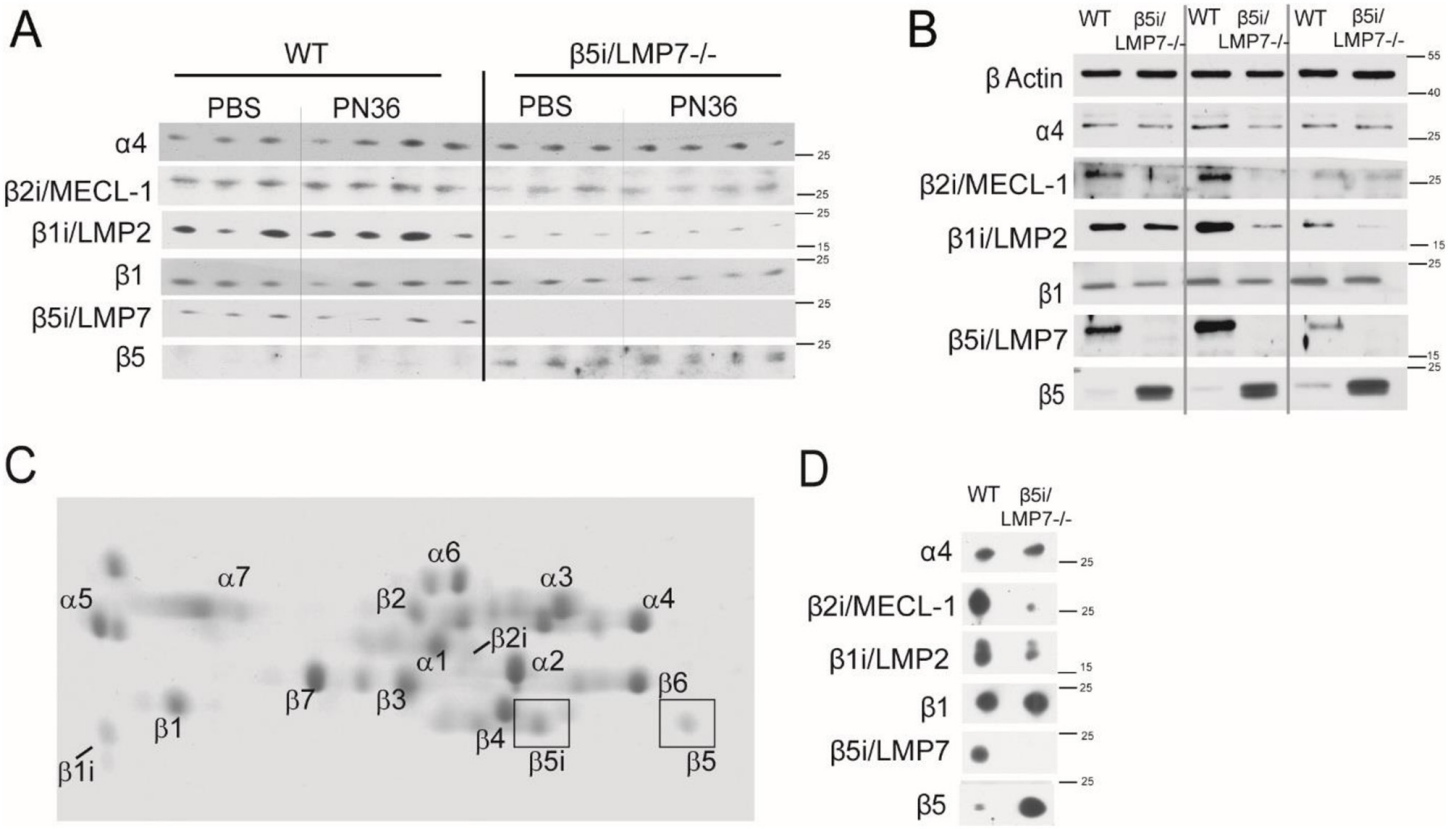
Both immuno- and standard subunits of the proteasome are constitutively expressed in leukocytes and liver. (A) Analysis of proteasome catalytic β-subunits expression on protein level in liver, 48 h after transnasal application of 5×10^6^ CFU PN36/mouse, performed by immuno-blot. (B) Analysis of proteasome catalytic β-subunits expression on protein level in BMM performed by immuno-blot (each group with n = 3). (C) 2D gel electrophoresis of purified 20S proteasomes, isolated from WT BMM. Proteasome subunits were visualized by coomassie stain. (D) Determination of β-subunits, incorporated in BMM 20S, detected by immuno-blot.

Because the observed diminished expression of the investigated opsonizing molecules in liver, lung, and macrophages of β5i/LMP7^-/-^ mice upon *S*. *pneumoniae* infection or co-cultivation was not the consequence of cytotoxic effects, we next asked whether it was the result of proteasome affected gene regulation provoked by altered proteasome function in consequence of changes in proteasome composition [9,12]. In agreement with previous reports [38–40], analysis of the proteasome subunit expression by immuno-blotting revealed that in WT liver, in addition to the β1 and β2 standard subunits, all three immuno-subunits were expressed (Fig 9A). We failed to detect substantial protein levels of the β5 standard subunit, suggesting that the liver of WT mice contains mixed-type proteasomes bearing the β5i/LMP7 subunit together with one or two of the β1 and/or β2 standard subunits. In the liver of β5i/LMP7^-/-^ mice, standard β5-subunit levels were detectable whereas the amount of the β1-subunit remained unchanged. In line with the importance of β5i/LMP7 for i-proteasome assembly, livers of β5i/LMP7^-/-^ mice revealed weaker signals for β1i/LMP2 and β2i/MECL-1. However, as indicated by the equal levels of the α4 subunit in WT and β5i/LMP7^-/-^ mice, the overall amount of proteasomes did not change upon β5i/LMP7 deficiency. Importantly, and as indicated by unaltered signals for all analyzed β-subunits, infection neither changed the proteasome subunit composition in WT nor in β5i/LMP7^-/-^ liver. Similar results were obtained by screening the subunit protein expression in uninfected BMM (Fig 9B). These results suggested that in macrophages or hepatocytes the diminished expression of immune modulators was associated to the absence of β5i/LMP7 and, namely, with reduced levels of i-proteasomes. To thoroughly study the influence of β5i/LMP7 deficiency in more detail, we analyzed proteasome subunit composition by 2D gel electrophoresis of purified 20S BMM proteasomes (Fig 9C). As shown in Fig 9D, WT macrophages assembled immuno- and standard subunits, whereas β5i/LMP7^-/-^ macrophages mainly exhibited standard proteasomes. Densitometrical analysis of β5 and β5i/LMP7 protein spots showed that macrophages expressed approximately 40% β5 and approximately 60% β5i/LMP7. In β5i/LMP7^-/-^ 20S proteasomes, the lack of the β5i/LMP7 protein was accompanied by an increased incorporation of the standard β5-subunit. In agreement with previous reports [41], the absence of β5i/LMP7 had a negative impact on the amount of β1i/LMP2 and β2i/MECL-1 subunits whose incorporation into 20S proteasomes was markedly reduced (Fig 9D). Both of the WT and β5i/LMP7^-/-^ BMM proteasomes presented equal tryptic- and caspase-like activities (S2 Fig), thereby confirming that the amounts of 20S complexes between samples were similar. By contrast, the chymotryptic-like activity of β5i/LMP7^-/-^ BMM proteasomes was by far superior to that detected in WT BMM proteasomes (S2 Fig). This is, however, in line with previous work showing that β5i/LMP7 deficiency results in an approximately 3-fold increase of the Suc-LLVY-MCA hydrolyzing activity [42].

### Deficiency in β5i/LMP7 is accompanied by modified intracellular signaling in stimulated macrophages

**Fig 10:**
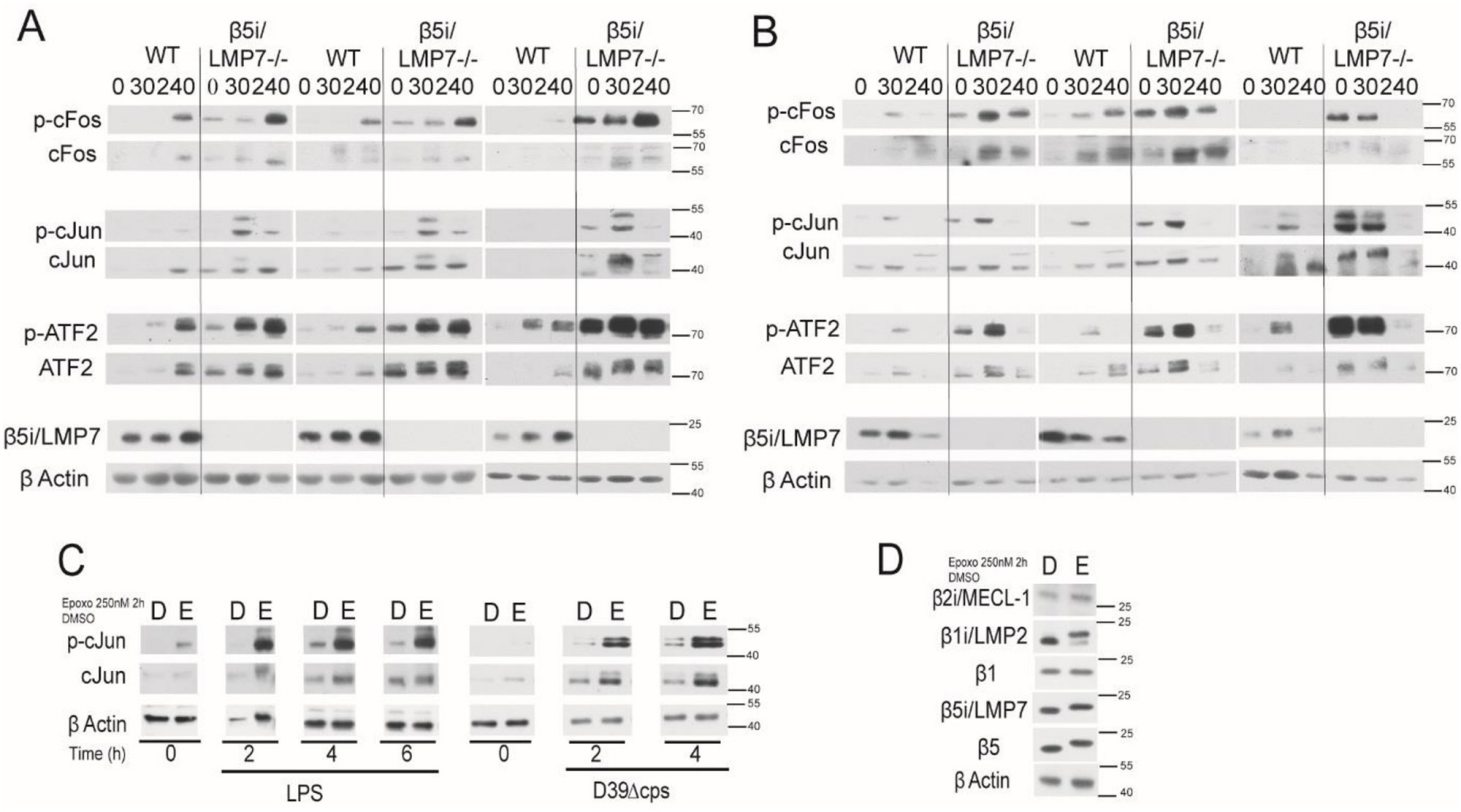
β5i/LMP7 deficiency as well as proteasome inhibition enhances AP-1 activation in stimulated macrophages. (A-B) Immuno-blot on phosphorylated and non-phosphorylated members of AP-1 and the NF-κB activating pathway in WT and β5i/LMP7^-/-^ BMM, stimulated *in vitro* for 0 min, 30 min, and 240 min with (A) LPS (1 μg/ml) or (B) D39Δcps (MOI 10) (each group with n = 3). (C) Immuno-blot on phosphorylated and non-phosphorylated cJun in RAW 264.7 cells, pretreated with DMSO or epoxomicin (250 nM) for 2 h, and subsequently stimulated with LPS (1 μg/ml) or D39Δcps (MOI 10) for 0 h, 2 h, 4 h, and 6 h. (D) Immuno-blot on catalytic β-subunits of the proteasome in RAW 264.7 cells incubated for 2 h with DMSO or epoxomicin (250 nM). Molecular shift indicates inhibitor binding.

Co-cultivation of epithelial cell with *S*. *pneumoniae* results in the induction of the MAP kinase pathway. This consequently initiates the phosphorylation and activation of the nuclear factor AP-1 regulating transcription of immune modulatory genes [43]. To study the influence of β5i/LMP7 deficiency on AP-1 activation we investigated its components, cJun, cFos, and ATF-2, together with their phosphorylated forms in LPS stimulated or *S*. *pneumoniae* co-cultivated BMM by immuno-blot (Fig 10A and 10B). LPS stimulation or *S*. *pneumoniae* exposure resulted in a robust phosphorylation of cJun, cFos, and ATF-2, which was more pronounced in β5i/LMP7^-/-^ macrophages compared to WT macrophages. Even in the absence of stimulation, β5i/LMP7^-/-^ macrophages expressed higher levels of phosphorylated and unphosphorylated cJun, cFos, and ATF-2 than their WT counterparts. Upon pathogen recognition, the protein expression of all three AP-1 components was up-regulated in macrophages of both genotypes. Of note, inhibition of proteasomes by epoxomicin resulted in AP-1 activation following stimulation of macrophages (Fig 10C). In this experiment, epoxomicin mainly inhibited the chymotrypsin-like activity of the proteasome by binding to β5, β5i/LMP7, and β1i/LMP2, but not to β1 and β2i/MECL-1, as indicated by a molecular upward shift in immuno-blot (Fig 10D). Macrophages, treated with epoxomicin, exhibited an enhanced protein level of cJun exposing a stronger phosphorylation signal. Following LPS stimulation or *S*. *pneumoniae* co-cultivation cJun protein expression and phosphorylation was induced, albeit to a more pronounced extent in epoxomicin pretreated cells. As previously reported [44], proteasome inhibition was accompanied by a reduced capacity of these cells to induce IL-1-β gene expression in response to LPS (S3 Fig). Likewise, epoxomicin-treated RAW 264.7 cells showed a much stronger down-regulation of *fcna* following LPS stimulation than those exposed to DMSO (S3 Fig). This is in line with our previous observation that β5i/LMP7 deficiency aggravates the drop of *fcna* mRNA levels upon infection (Fig 7C). These results suggest that changes in proteasome composition or inhibition had an effect on transcription factor activation which, in turn, influences opsonin gene expression.

## Discussion

The *S*. *pneumoniae* infection model used in this study is a model of untreated fatal pneumonia. Macrophages, neutrophils, and lymphocytes failed to clear pneumococci, which proliferated and induced bacteremia in addition to the initial pulmonary infection. All mice developed a sequential pathogenesis with a lethal outcome. However, mice deficient in the β5i/LMP7 subunit and hence in fully functional i-proteasomes developed an aggravated systemic inflammatory response with enhanced bacteremia likely due to a diminished expression of opsonizing molecules.

Clinical signs and diagnostic markers, such as body weight loss and SAA expression were significantly more prominent in β5i/LMP7^-/-^ animals, suggesting a more severe course of pneumococcal disease with critical illness [32]. Although, there was no difference in the overall approximate survival rate between infected WT and β5i/LMP7^-/-^ mice, the latter showed a markedly aggravated pneumococcal pneumonia with an advanced chronology of microbiological and immunological processes. β5i/LMP7 deficient mice developed a state of relative systemic immune suppression during the late phase of pneumonia, with reduced amounts of circulating granulocytes. In addition, the total amount of leukocytes significantly declined in β5i/LMP7^-/-^ mice resulting in prominent leukopenia. This was established by a significant lymphopenia and correlated with the magnitude of illness. Our results are in line with previous reports that defined the final pathogenesis step of a fatal pneumococcal pneumonia with a systemic inflammatory response syndrome which transits into a relative systemic immune suppression, manifested by decreased levels of circulating leukocytes including neutrophils and lymphocytes [45].

β5i/LMP7^-/-^ mice developed a significantly increased pneumococcal burden in blood during the late phase of pneumonia. Although intracellular killing properties were not affected in β5i/LMP7^-/-^ leukocytes, *S*. *pneumoniae* stimulated β5i/LMP7^-/-^ macrophages released reduced levels of NO, which is known to control pneumococcal viability [37]. These results are in accordance with those of Reis et al., reporting diminished NO production in β5i/LMP7^-/-^ macrophages stimulated with LPS [46]. Although Bergeron et al. documented a sustained release of NO in BAL fluid during the late phase of pneumonia [45], we were not able to detect NO release in BAL fluid of either WT or β5i/LMP7^-/-^ mice, and thus were not able to demonstrate a relationship between NO release and bacterial burden.

Opsonizing molecules such as pentraxins and complement components are known to promote elimination of invading bacteria [47]. Our data show that infection was accompanied by the up-regulation of *c1qa* and *ptx3* as well as the down-regulation of *mbl2* (Fig 7). Remarkably, the induction *ptx3* gene expression was significantly less pronounced in β5i/LMP7^-/-^ mice than in WT animals, while the decreased expression of *mbl2* was more prominent in β5i/LMP7-deficient mice, when compared to their WT littermates. In addition, *fcna* was found to be substantially repressed following infection in β5i/LMP7^-/-^ mice but not in WT animals. Altogether, these data highlight the fact that the amount of opsonizing molecules during infection is far more limited in β5i/LMP7^-/-^ mice. The observation that β5i/LMP7^-/-^ mice exhibit an aggravated systemic dissemination of *S*. *pneumoniae* upon infection is in full agreement with the observed pathological phenotypes of infected opsonin deficient individuals. PTX3^-/-^ mice were highly susceptible towards lung infection with Pseudomonas aeruginosa [48]. In animal models, human CRP protects against a lethal infection with *S*. *pneumoniae*, and in humans, it contributes to host defense during bacteremic pneumonia [49]. The C1q-mediated complement activation pathway was shown to be required for an effective innate immune response against *S*. *pneumoniae* [50]. Ficolin A is thought to be the key recognition component of the lectin activation pathway, essential for an effective host defense against pneumococcal infection [51]. Furthermore, patients, homozygotes for an MBL codon variant exhibited reduced MBL serum levels and were highly susceptible to invasive pneumococcal infections [26]. Surprisingly, the induction of PAFR during infection with *S*. *pneumoniae* was slightly impaired in β5i/LMP7^-/-^ mice (S1 Fig). Given the role of PAFR as a virulence factor receptor, a decreased susceptibility of the animals could result, which is not the case. However, this apparent discordance can be easily explained by the fact that β5i/LMP7^-/-^ mice express substantially less opsonizing molecules which may overcome the potential beneficial effect of decreased expression of PAFR on the disease.

Importantly, Weber et al. have shown that the transporter with antigen processing (TAP) and the β2-microglobulin (β2-m) were dispensable for the clearance of *S*. *pneumoniae* [52]. This suggests that MHC class I antigen processing and presentation is not required in this process and underscores the importance of the β5i/LMP7 subunit in innate immunity in the context of *S*. *pneumoniae* infection by regulating gene expression of critical mediators including opsonins. The β5i/LMP7 subunit deficiency has a substantial impact on the assembly and composition of proteasomes in cells constitutively expressing or up-regulating i-proteasomes. Changes in the cellular proteasome sub-type composition lead to altered proteasomal activities affecting cell and tissue functions during infection and inflammation [7–9,12,53]. In our *S*. *pneumoniae* infection model, the altered proteasome composition and its presumably impaired function in macrophages and liver of β5i/LMP7^-/-^ mice coincided with affected gene transcription of immune modulating molecules. It has been shown that β5i/LMP7 deficiency negatively affects the incorporation of β1i/LMP2 and β2i/MECL1 and hence the overall proteasome sub-type composition of cells [41]. Dahlmann et al. showed that different proteasome sub-types exhibited altered enzymatic properties, differing in their substrate degradation rate and in the quality of generated products [6]. In fact, the presence and absence of the β5i/LMP7 subunit in 20S proteasomes was shown to affect the structure as well as the function of proteasomes [5,54]. Therefore, it is conceivable that different proteasome sub-types may influence gene transcription by differentially degrading specific proteins of intracellular signaling cascades or by degrading regulatory molecules that either inhibit or activate gene transcription.

The role of proteasomes and i-proteasomes in the regulation of pro-inflammatory cytokines via NF-κB is well described [7,9,12,53]. Here, we analyzed the effect of i-proteasome deficiency on the *S*. *pneumoniae* induced activation of AP-1 which is believed to regulate inflammatory-mediated gene transcription [19]. Gerber et al. reported that proteasome inhibition induced MAPK activation and subsequent cJun-dependent AP-1 activity in monocytes [55]. Our experiments showed that β5i/LMP7 deficiency confers an enhanced effect on AP-1 activation. This suggests that structural changes induced on the proteasome complex due to β5i/LMP7 deficiency which concomitantly influence the proteolytic properties of the proteasome most likely alter signaling pathways. Enhanced AP-1 activation may be the consequence of diminished degradation of up-stream kinases, such as IRAK-1 or MAPKs [56]. Because i-proteasomes have an enhanced ability to degrade oxidized, misfolded, and poly-ubiquitinated proteins [7], it is also conceivable that AP-1 activation may be facilitated by enhanced proteotoxic and oxidative stress due to β5i/LMP7 deficiency [57].

Pathogen activated signaling pathways are complex and numerous nuclear factors have been demonstrated to play critical roles in the subsequent expression of inflammatory mediators. However, regarding the complexity of the intracellular signaling, redundancy among signaling cascades, influencing and compensating each other, is likely. It is therefore difficult to clarify a specific impact of β5i/LMP7 deficiency on gene regulation. However, the regulated diversity of proteasome sub-types is most likely important to precisely adjust endogenous cell signaling and activity of transcription factors adapted for cell specific functions.

In conclusion, the absence of the catalytic immuno-subunit β5i/LMP7 in murine macrophages and liver tissue changes the composition of their proteasomes that may have modified pathogen induced intracellular signaling pathways resulting in reduced gene transcription of immune modulating molecules. In our study, opsonins are prime examples of immune molecules whose expression is substantially impaired following infection in i-proteasomes-free mice. The diminished opsonin expression may result in an aggravated systemic dissemination of *S*. *pneumoniae* in β5i/LMP7^-/-^ mice. The impaired bacterial elimination is accompanied by aggravated pneumonia with early mortality in consequence of critical illness during the late phase of disease.

## Supporting Information

S1 Fig β5i/LMP7 deficiency diminishes PAFR induction upon infection with *S*. *pneumoniae*. PAFR gene expression analysis of lungs, 48 h after transnasal application of PBS or 5x10^6^ CFU PN36/mouse, as indicated and performed by real-time PCR (each group with n = 5–8; statistical analysis by Student’s *t* test. *p<0.05 and **p<0.001). The amount of transcripts for each gene was normalized to the HPRT1 housekeeping gene. Given are the *ΔCt* measured values.
(TIF)

S2 Fig Enzymatic activities of BMM 20S proteasomes isolated from wild-type and β5i/LMP7^-/-^ mice. One hundred and twenty five nanograms of purified BMM 20S proteasomes isolated from wild-type and β5i/LMP7 mice was incubated with each of the Suc-LLVY-AMC (100 μM, chymotryptic-like), Bz-VGR-AMC (200 μM) and z-LLE-AMC (200 μM) to monitor the chymotryptic-, tryptic- and caspase-like activity, respectively. After 40 min of incubation at 37°C, the plates were read at an absorption/emission of 360/460 using a microplate fluorescence reader. Shown is one representative experiment out of three: ***p<0.05 (Student’s *t*-test).
(TIF)

S3 Fig Proteasome inhibition is accompanied by decreased expression of fcna in RAW cells following LPS stimulation. RAW 264.7 cells were subjected to a 2-h treatment with 250 nM epoxomicin (or DMSO as a control) prior to a stimulation with 1 μg/ml LPS for 24 h. Cells were collected at 0, 2, 4, 6, 8 and 24 h post-stimulation for RNA extraction. One microgram of total RNA was used for reverse transcription (RT) and quantitative PCR analysis of IL-1-β and *fcna*, as indicated. After real-time PCR, the amount of transcripts for each gene was normalized to the HPRT1 housekeeping gene. Given are the *ΔCt* measured values. One representative experiment out of two is shown. *p<0.1 and **p<0.05 versus cells exposed to DMSO (Student’s *t*-test).
(TIF)
