# Supplementary figures and images for: Proteasome β5i Subunit Deficiency Affects Opsonin Synthesis and Aggravates Pneumococcal Pneumonia
Source: PLoS One. 2016 Apr 21;11(4):e0153847. doi: 10.1371/journal.pone.0153847 (PMC4839637; doi:10.1371/journal.pone.0153847)

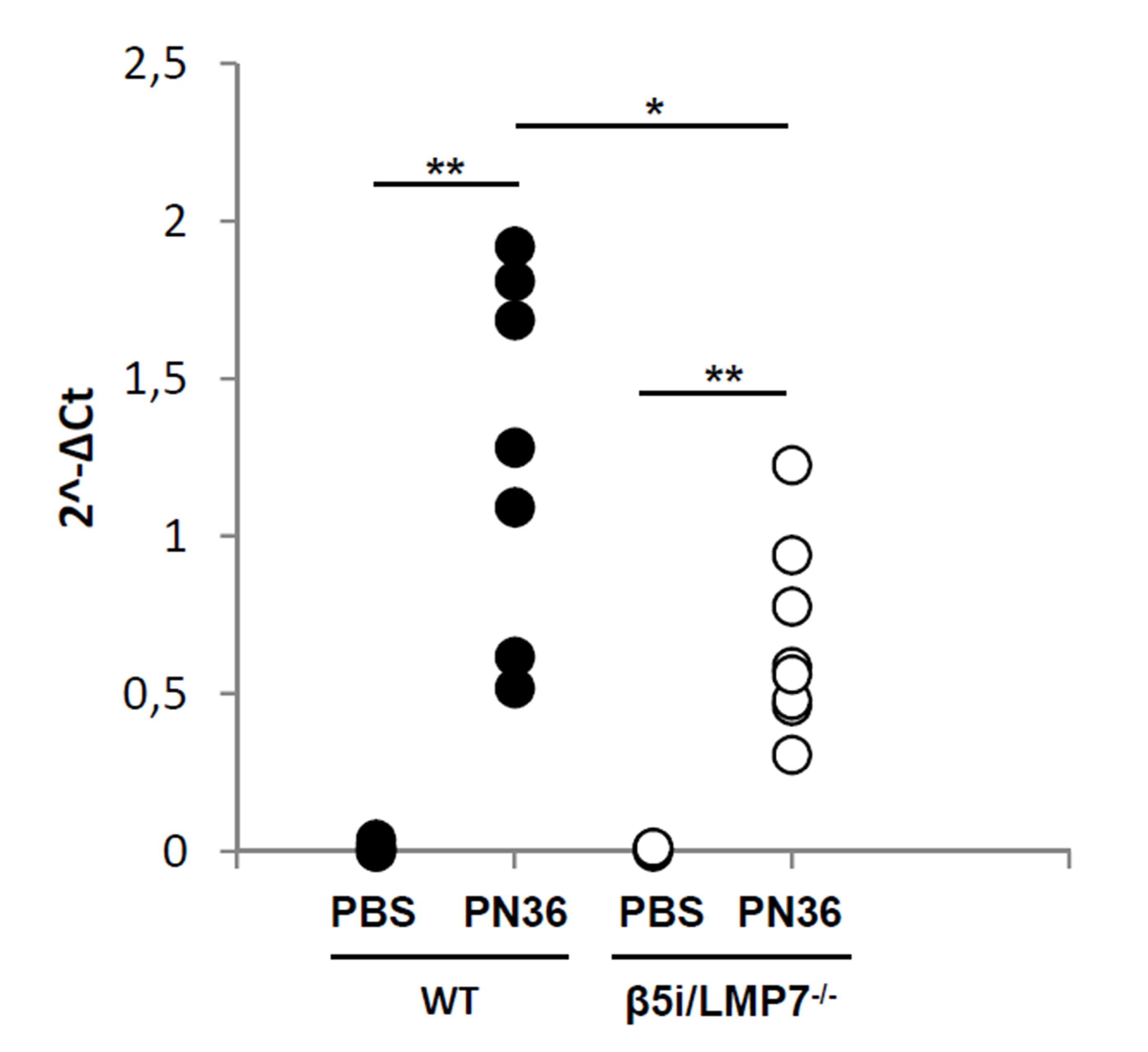

Supplement: S1 Fig — PAFR gene expression analysis of lungs, 48 h after transnasal application of PBS or 5x10^6^ CFU PN36/mouse, as indicated and performed by real-time PCR (each group with n = 5–8; statistical analysis by Student’s t test. *p<0.05 and **p<0.001). The amount of transcripts for each gene was normalized to the HPRT1 housekeeping gene. Given are the ΔCt measured values. (TIF) [file pone.0153847.s001.tif]

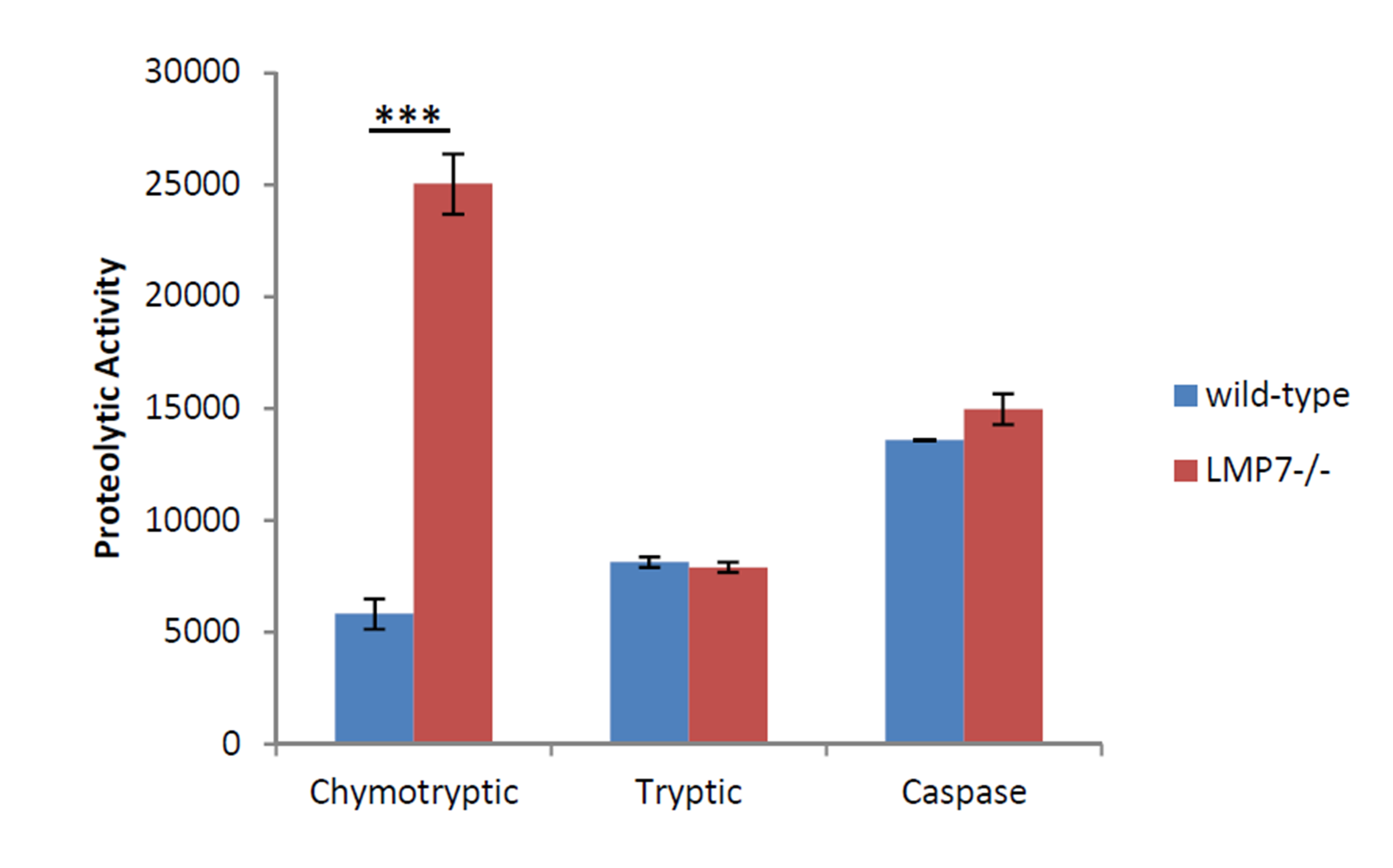

Supplement: S2 Fig — One hundred and twenty five nanograms of purified BMM 20S proteasomes isolated from wild-type and β5i/LMP7 mice was incubated with each of the Suc-LLVY-AMC (100 μM, chymotryptic-like), Bz-VGR-AMC (200 μM) and z-LLE-AMC (200 μM) to monitor the chymotryptic-, tryptic- and caspase-like activity, respectively. After 40 min of incubation at 37°C, the plates were read at an absorption/emission of 360/460 using a microplate fluorescence reader. Shown is one representative experiment out of three: ***p<0.05 (Student’s t-test). (TIF) [file pone.0153847.s002.tif]

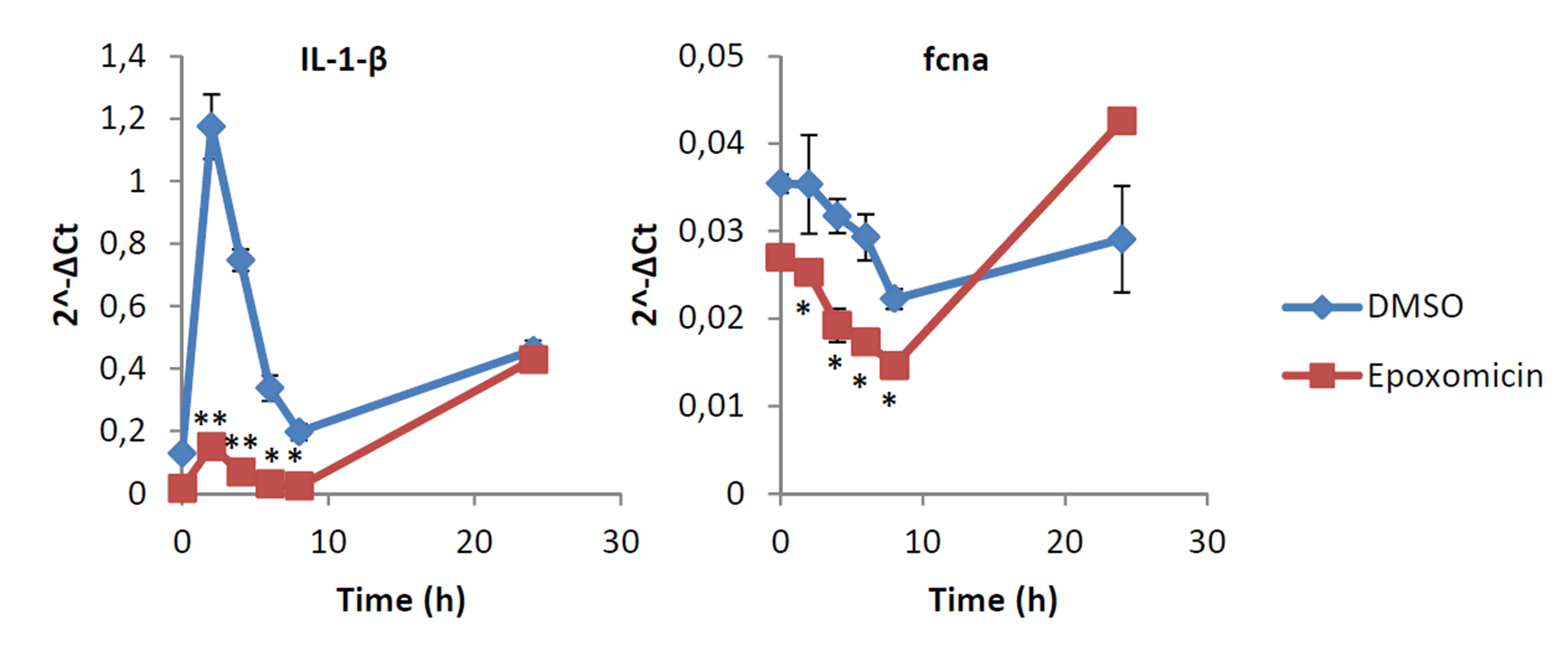

Supplement: S3 Fig — RAW 264.7 cells were subjected to a 2-h treatment with 250 nM epoxomicin (or DMSO as a control) prior to a stimulation with 1 μg/ml LPS for 24 h. Cells were collected at 0, 2, 4, 6, 8 and 24 h post-stimulation for RNA extraction. One microgram of total RNA was used for reverse transcription (RT) and quantitative PCR analysis of IL-1-β and fcna, as indicated. After real-time PCR, the amount of transcripts for each gene was normalized to the HPRT1 housekeeping gene. Given are the ΔCt measured values. One representative experiment out of two is shown. *p<0.1 and **p<0.05 versus cells exposed to DMSO (Student’s t-test). (TIF) [file pone.0153847.s003.tif]
